# Deficient Resident Memory T Cell and CD8 T Cell Response to Commensals in Inflammatory Bowel Disease

**DOI:** 10.1093/ecco-jcc/jjz175

**Published:** 2019-10-26

**Authors:** Alistair Noble, Lydia Durant, Lesley Hoyles, Anne L Mccartney, Ripple Man, Jonathan Segal, Samuel P Costello, Philip Hendy, Durga Reddi, Sonia Bouri, Dennis N F Lim, Toby Pring, Matthew J O’Connor, Pooja Datt, Ana Wilson, Naila Arebi, Ayesha Akbar, Ailsa L Hart, Simon R Carding, Stella C Knight

**Affiliations:** 1 Gut Microbes and Health Programme, Quadram Institute Bioscience, Norwich, UK; 2 Antigen Presentation Research Group, Imperial College London, Northwick Park and St Mark’s Campus, Harrow, London, UK; 3 Department of Surgery and Cancer, Imperial College London, South Kensington Campus, London, UK; 4 Department of Bioscience, Nottingham Trent University, Nottingham, UK; 5 Department of Food and Nutritional Sciences, University of Reading, Reading, UK; 6 St Mark’s Hospital, London North West University Healthcare NHS Trust, Harrow, UK; 7 Department of Gastroenterology, Queen Elizabeth Hospital, Adelaide, SA, Australia; 8 Norwich Medical School, University of East Anglia, Norwich, UK

**Keywords:** Dendritic cells, microbiota, T lymphocytes

## Abstract

**Background and Aims:**

The intestinal microbiota is closely associated with resident memory lymphocytes in mucosal tissue. We sought to understand how acquired cellular and humoral immunity to the microbiota differ in health versus inflammatory bowel disease [IBD].

**Methods:**

Resident memory T cells [Trm] in colonic biopsies and local antibody responses to intraepithelial microbes were analysed. Systemic antigen-specific immune T and B cell memory to a panel of commensal microbes was assessed.

**Results:**

Systemically, healthy blood showed CD4 and occasional CD8 memory T cell responses to selected intestinal bacteria, but few memory B cell responses. In IBD, CD8 memory T cell responses decreased although B cell responses and circulating plasmablasts increased. Possibly secondary to loss of systemic CD8 T cell responses in IBD, dramatically reduced numbers of mucosal CD8^+^ Trm and γδ T cells were observed. IgA responses to intraepithelial bacteria were increased. Colonic Trm expressed CD39 and CD73 ectonucleotidases, characteristic of regulatory T cells. Cytokines/factors required for Trm differentiation were identified, and in vitro-generated Trm expressed regulatory T cell function via CD39. Cognate interaction between T cells and dendritic cells induced T-bet expression in dendritic cells, a key mechanism in regulating cell-mediated mucosal responses.

**Conclusions:**

A previously unrecognised imbalance exists between cellular and humoral immunity to the microbiota in IBD, with loss of mucosal T cell-mediated barrier immunity and uncontrolled antibody responses. Regulatory function of Trm may explain their association with intestinal health. Promoting Trm and their interaction with dendritic cells, rather than immunosuppression, may reinforce tissue immunity, improve barrier function, and prevent B cell dysfunction in microbiota-associated disease and IBD aetiology.

## 1. Introduction

Large numbers of lymphocytes reside in the intestinal mucosa and play a key role in barrier function and immune surveillance. Immunity against infection is provided by long-lived memory T cells reactive to foreign antigens as well as antibody.^1^ Memory T cells can be broadly categorised into circulating [central memory and effector memory] and tissue-resident, non-recirculating cells called resident memory T cells [Trm].^2^ Trm provide potent barrier immunity in mucosal tissues due to their high motility, rapid reactivation of effector function, and ability to recruit further immune responses via cytokine [e.g. IFN-γ] secretion. However, the role of Trm in human disease is unclear, and how they interact with resident microbes that make up the intestinal microbiota is not understood.

Inflammatory bowel disease [IBD] is thought to be perpetuated by intestinal microbial dysbiosis leading to episodic colitis [ulcerative colitis, UC] or localised inflammation anywhere along the gastrointestinal [GI] tract [Crohn’s disease, CD], mediated by Th17 or other subsets of CD4 T cells.^3,4^ Disease aetiology involves interaction of multiple genetic susceptibilities with environmental factors, including diet and lifestyle factors, that can affect the microbiota. However, most studies to date have focused on sequence-based profiling of microbiomes in disease; how different microbial species interact with the immune system is not well understood. Mouse studies indicate the colonic microbiota is essential for recruiting sufficient CD4 Foxp3-expressing regulatory T cells [Treg] to the colon to prevent inflammation.^5,6^ This suggests that IBD results from a failure of Treg-mediated tolerance to commensals in the GI tract.

Here we studied memory T cell responses to a panel of intestinal commensal bacteria in IBD patients and healthy controls, and analysed Trm populations in the epithelium and lamina propria colonic tissue where they are in close proximity to mucosa-associated microbes. Our data show that underlying disease in human IBD is associated with reduced CD8 T cell responses to commensal bacteria leading to Trm deficiency in the colon, and chronic B cell activation and excess IgA secretion associated with a loss of barrier immunity. We also show that human Trm express Treg function, and propose specific mechanisms to explain how loss of Trm:dendritic cell interaction could contribute to the development of inflammatory disease.

## 2. Methods

### 2.1. Study design

The study aimed to determine the role of resident memory T cells in IBD. Donors [age 16–80] were recruited to the study from outpatient clinics of St Mark’s Hospital and included those with a diagnosis of CD or UC, and healthy donors undergoing investigative endoscopy. None of the CD patients had a history of obstruction, perianal disease, or ileitis alone. Patients were recruited over a fixed period determined by ethical permission; no data were excluded at the end of the study. Additional healthy blood donors were recruited from hospital staff and visitors. Ethics approval was obtained from the Health Research Authority UK and London Brent Research Ethics Committee. Written informed consent was received from participants before inclusion in the study.

### 2.2. Colonic intraepithelial lymphocytes [IEL], lamina propria lymphocytes [LPL], and intraepithelial microbe [IEM] isolation

Five left colon and five right colon biopsies [10 mg tissue each] were obtained from macroscopically non-lesional tissue sites at routine colonoscopy in all patients as described.^7^ IEL and IEM were released from biopsies using DTT/EDTA and harvested by centrifugation at 300 *g* [5 min]. IEM were obtained by centrifugation of resulting supernatants at 4500 *g* [20 min]. LPL were obtained by collagenase digestion of remaining tissue; all cells were phenotyped and counted by flow cytometry. Cells were washed in PBS and stained for viability using LIVE/DEAD Fixable-near-IR stain [ThermoFisher] before addition of surface-staining antibodies in fetal calf serum. In some cases cells were then fixed/permeabilised for intranuclear staining using the Foxp3 buffer set [ThermoFisher, as instructions]. Antibodies used are listed in Supplementary File 1, available as Supplementary data at *ECCO-JCC* online. All samples were analysed on a BD Biosciences FACS Canto II and data were analysed by FlowJo software [Tree Star], with volumetric sampling determined using Perfect Count microspheres^TM^ [Cytognos].

### 2.3. In vitro differentiation of Trm-like cells from human peripheral blood mononuclear cells

Naïve CD8 T cells were purified by magnetic selection from healthy donor peripheral blood mononuclear cells [PBMC] using the naïve CD8 T cell isolation kit [Miltenyi Biotec] and were >98% CD8^+^ and >98% CD45RA^+^. Naïve CD8 T cells were stimulated with plate-bound anti-CD3 [1 µg/ml], soluble anti-CD28 [1 µg/ml], and IL-2 [5 ng/ml, Peprotech]. Further additions of TGF-β [3 ng/ml, R&D Systems], IFN-β [10 ng/ml, R&D], all-trans retinoic acid [ATRA; 10 nM, Sigma], FICZ [AhR agonist; 100 nM, Tocris Bioscience] were made at the start of the 7-day culture. Cultured cells were washed in PBS, stained for viability and surface or intracellular markers as above. Tc1/Trm-like cells were analysed for cytokine production by re-stimulation with PMA [20 ng/ml] + ionomycin [400 ng/ml] + monensin [3 µM] for 4 h before staining using Foxp3 staining buffer set.

### 2.4. Commensal-specific T and B cell memory proliferative responses

Commensal species were isolated from the caecum of healthy donors with the exception of *Collinsella aerofaciens,* which was from faeces.^8–10^ Strains were grown anaerobically in Hungate tubes containing Wilkins-Chalgren broth [37°C for 24 h]. Aliquots [1 ml] were centrifuged [13 000 rpm for 10 min], supernatants removed, and cell pellets snap-frozen with dry ice before storage at -80°C. PBMC were obtained over Ficoll gradients and labelled with CellTrace Violet^TM^ [1 µM, Life Technologies] according to manufacturer’s instructions, then cultured at 4 × 10^6^/ml in XVIVO15 serum-free medium (Lonza, + 50 µg/ml gentamycin [Sigma] and penicillin/streptomycin [Life Technologies, 1/100]); 2 × 10^5^ killed bacteria from 19 species [as in Figure 5 below] were added to 0.2-ml cultures, and microbe-specific CD4^+^/CD8^+^ T cell and B cell responses were determined after 7 days’ culture. Cultured cells were analysed by staining with LIVE/DEAD stain, CD4/CD8/CD19/integrin-β7/CLA/CD39.

### 2.5. Suppression assays

A fraction of healthy donor PBMC were cryopreserved before isolation of naïve CD8 T cells and differentiation into Tc1- or Trm-like cells, as described above. Cells were cultured at 0.5 × 10^6^/ml in 0.4-ml cultures; Tc1 cells were generated with anti-CD3/28+IL-2 only and Trm-like cells with addition of TGF-β, IFN-β, ATRA, and FICZ. After 7 days cells were washed and autologous PBMC thawed, before labelling with CellTrace Violet^TM^. Labelled target cells were cultured in U-bottom wells [0.2-ml XVIVO-15] at 10^6^/ml with or without addition of unlabelled Tc1/Trm cells and CD39 inhibitor ARL67156 [200 µM, Tocris Bioscience]. Cells were stimulated by addition of SEB [0.1 µg/ml, Sigma] and stained after 4 days with LIVE/DEAD stain, CD3/CD4/CD8/CD25. Cells were gated for CellTrace Violet^+^ CD8^+^ T cells, and fractions of cells which had divided and upregulated CD25 assessed.

### 2.6. Induction of transcription factors and cytokines in DC

PBMC from healthy donors were cultured at 10^6^/ml in RPMI-1640 medium [Sigma] supplemented with 10% newborn calf serum [Sigma] and antibiotics as above. LPS [1 µg/ml], SEB [10 ng/ml, both Sigma], and anti-IFN-γ [50 µg/ml] or isotype control IgG1 were added. After overnight culture all cells were stimulated with LPS + poly I:C [1 µg/ml, Sigma] + monensin [3 µM] for a further 4 h and stained for lineage markers, HLA-DR/CD123/CD11c/T bet/TNF-α /IFN-α and LIVE/DEAD stain using Foxp3 buffer set. Gating for singlet mDC and pDC was performed as shown in Supplementary File 1. Strict gating for CD11c-negative cells was used to exclude mDC precursors from the pDC gate—this was confirmed by lack of staining for CD33, CX3CR1, and Axl.

### 2.7. Measurement of antibody responses

IEM were labelled with SYBR Green DNA stain [Life Technologies, 1/100 000], anti-IgA-APC/anti-IgG-APC/Cy7, and analysed by flow cytometry to determine proportion [%] of bacteria coated with antibodies in the gut. Circulating antibodies to commensal species were determined by incubation of plasma [1/10 in 0.1% BSA, 0.5 ml] with 1 × 10^5^ bacteria [30 min], followed by centrifugation [12 000 *g,* 10 min] and staining as for IEM [or isotype controls for each sample]. Intact microbes were gated according to SYBR Green, and ratio of geometric mean fluorescence intensity of staining for test sample vs isotype control was used as measure of antibody titre. Plasma IgG antibodies to viruses were measured using ELISA kits from Abcam according to instructions.

### 2.8. Statistical analysis

GraphPad Prism 7 software [GraphPad, San Diego, CA] was used to plot and analyse the data. Clinical data were analysed by one-way ANOVA or, where populations were skewed, Kruskal-Wallis tests. For in vitro experiments, data were analysed using two-tailed paired t tests or one-way ANOVA for multiple experimental conditions; *p*-values less than 0.05 were considered significant and indicated by: *:*p* < 0.05; **:*p* < 0.01; ***:*p* < 0.001.

## 3. Results

### 3.1. Human colonic Trm are identified by CD103 and Runx3 and express Treg markers CD39 and CD73

**Figure 1.**
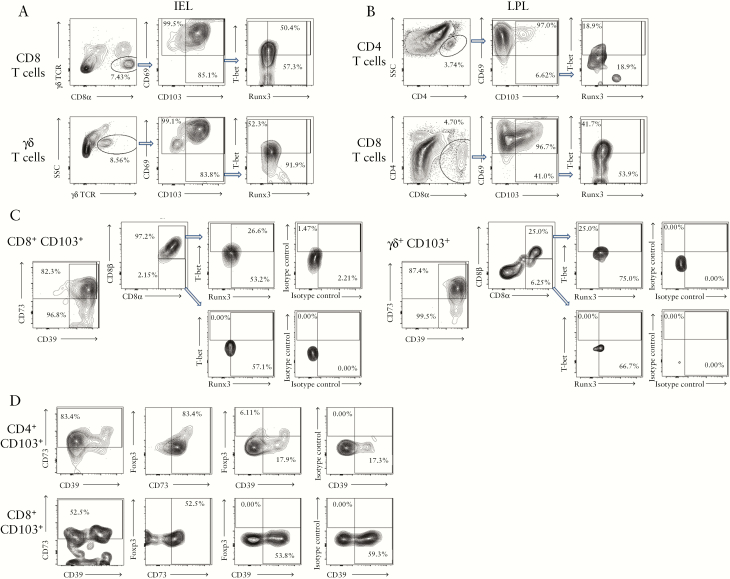
Human colonic Trm are identified by CD103 and express Runx3, T-bet, and regulatory T cell markers but not Foxp3. A: CD8 T cell and γδ T cell populations were identified in IEL fractions and stained for CD69/CD103 surface Trm markers; gated CD103^+^ cells were stained for intranuclear Runx3 and T-bet. B: CD4 and CD8 T cell populations were identified in LPL fractions and stained for CD69/CD103; gated CD103^+^ cells were stained for Runx3 and T-bet. C: IEL CD8^+^ and γδ T cell populations were stained for CD39/CD73 Treg-associated ectonucleotidases and CD8αβ to distinguish conventional vs innate-type lymphocytes. CD8αβ and CD8αα subsets were separately gated and T-bet and Runx3 expression shown, including isotype control staining for transcription factors. D: LPL CD4^+^ and CD8^+^ Trm-like populations were stained for surface CD39/CD73 and intranuclear Foxp3. Right panels show isotype control for Foxp3 stain. Staining is from right colon biopsies of healthy donors and is representative of at least five individual donors. Similar data were obtained in left colon. IEL, intraepithelial lymphocytes; LPL, lamina propria lymphocytes.

To evaluate the role of Trm in IBD, we first identified Trm in intraepithelial lymphocytes [IEL] and lamina propria lymphocytes [LPL] from healthy control [HC], CD, and UC colonic biopsies [non-inflamed tissue; clinical and demographic patient characteristics are shown in Tables 1–3]. In healthy IEL [Figure 1a], all T cells including CD8^+^ or γδ T cells expressed the CD69 putative Trm marker. However, CD103 distinguished Trm from effector memory T cells, which was confirmed by their intranuclear expression of the Runx3 transcription factor which controls the Trm transcriptional programme in mice.^11^ A fraction of Trm expressed T-bet, which controls the Th1 programme of differentiation.^12^ In LPL [Figure 1b], main populations were CD4^+^ and CD8^+^ T cells, and the latter not only contained a much larger proportion of CD103^+^ Trm-like cells but also had higher levels of Runx3 and T-bet than their CD4 counterparts. IEL lacked CD4 cells and no γδ T cells were found in LPL. Trm and γδ T cells expressed high levels of the Treg markers CD39 and CD73, suggesting immunosuppressive function [Figure 1c and d]. CD8 Trm were nearly all conventional T cells expressing CD8αβ heterodimer [Figure 1c] and no Foxp3 transcription factor [Figure 1d]. The vast majority of cells expressing CD39 and CD73 were also Foxp3^-^ [barring a small fraction of the CD4 Trm], suggesting that the Trm themselves contribute to maintaining tissue homeostasis.

**Table 1.** Clinical characteristics of St Mark’s Hospital colonoscopy patients donating colonic biopsies.

Characteristic	HC	CD	UC
n	23	11	18
Male/female	13/10	4/7	12/6
Median age [95% CI] at sampling	51.5 [41–57]	43 [28–57]	53.5 [47–60]
Median age [95% CI] at diagnosis		26 [18–47]	35 [29–42]
Inflammation: endoscopic subscores for UC:			
Mayo 0			8
Mayo 1			3
Mayo 2			6
Mayo 3			1
IBD medications at sampling:			
Aminosalicylates		4	15
Azathioprine/6-mercaptopurine		4	1
Buscopan		1	0
Adalimumab		1	0
None		4	3
Non-IBD medications at sampling:			
Metformin/gliclazide/statin		0	2
Ondansetron		1	0
Certirazine		1	0
None		9	16

**Figure 2.**
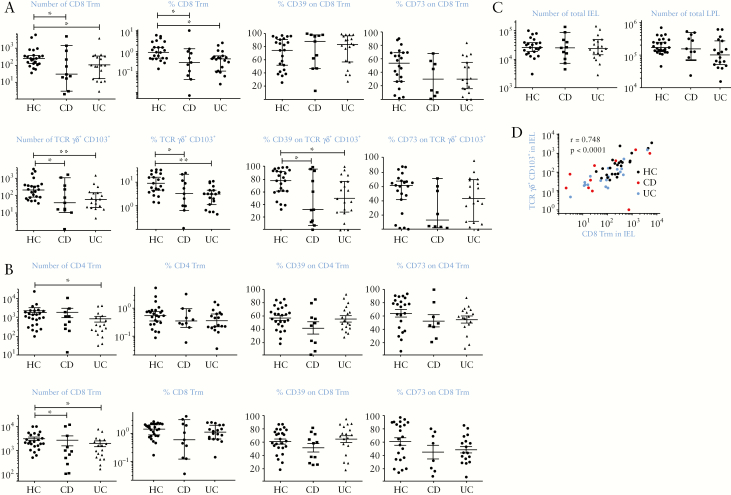
Quiescent IBD is associated with reduced numbers of Trm and γδ T cells in colonic tissue. A: Numbers and percentages of CD8^+^ γδ TCR^-^ CD103^+^ Trm recovered from IEL [upper graphs] and γδ CD103^+^ T cells in IEL [lower graphs], alongside CD39 and CD73 expression on these populations. B: Numbers and percentages of CD4^+^ CD103^+^ Trm [upper graphs] and CD8^+^ CD103^+^ Trm [lower graphs] recovered from LPL, alongside CD39 and CD73 expression. Full gating strategies are shown in supplementary methods. C: Total live cell numbers in IEL and LPL fractions [including epithelial cells]. D: Correlation of CD8 Trm and γδ T cell numbers in IEL populations from right colon biopsies of all donors. HC: healthy controls, *n* = 25; CD: Crohn’s disease, *n* = 12; UC: ulcerative colitis, *n* = 20. Median values ± 95% confidence intervals are shown; statistically significant differences between groups [Kruskal–Wallis test] are indicated. Spearman correlation coefficient was calculated in D. IEL, intraepithelial lymphocytes; LPL, lamina propria lymphocytes; IBD, inflammatory bowel disease.

*Demographic and clinical data analysed in* Figures 2, 4d, and Supplementary Figure S1, available as Supplementary data at *ECCO-JCC* online.

HC, healthy controls; CD, Crohn’s disease; UC, ulcerative colitis; CI, confidence interval.

**Table 2.** Clinical features/Montreal classification of CD patients donating colonic biopsies.

Donor	Age at diagnosis	Disease location	Disease behaviour	Inflammation
1	*A2*	L3	B1	None
2	A3	L3	B1	Mucosal erosions, polyps
3	A2	L2	B1	Patchy area of inflammation, polyps
4	A1	L0	B1	None
5	A2	L3	B1	Small mucosal ulceration
6	A2	L3	B1	Ulceration, scarred colon
7	A3	L3	B1	Ulceration
8	A2	L0	B1	None
9	A2	L0	B1	None
10	A2	L0	B1	None
11	A2	L3	B1	Mild inflammation/ulceration

*Demographic and clinical data analysed in* Figures 2, 4d, and Figure S1, available as Supplementary data at *ECCO-JCC* online.

**Table 3.** Clinical characteristics of St Mark’s Hospital blood donors and healthy volunteers.

Characteristic	HC	CD	UC
n	18	17	14
Male/female	13/5	8/9	7/7
Median age [95% CI] at sampling	41.5 [30–54]	43.5 [33–61]	54.5 [35–63]
Median age [95% CI] at diagnosis		25 [20–44]	38.5 [25–47]
Symptoms at sampling:			
Diarrhoea/loose stools		1	2
Occasional loose motion/watery stool		2	1
A bdominal pain		2	1
Perianal pain/itch/disease		2	1
Lethargy		2	0
Proctitis		0	1
None		9	8
IBD medications at sampling:			
Aminosalicylates		8	8
Azathioprine/6-mercaptopurine		8	2
Corticosteroids		1	0
Vedolizumab		1	0
Antibiotics		1	0
Methotrexate		1	0
None		1	6
Non-IBD medications at sampling:			
Vitamins D/D3/B12/multi		4	3
Metformin/gliclazide		0	2
Statins		1	2
Hydroxychloroquine		0	1
Proton pump inhibitor		0	1
Alendronate		1	0
Finasteride		1	0
Loperamide		1	1
None		12	9

*Demographic and clinical data analysed in* Figures 4, 5, amd Figs S2, S3, S4, available as Supplementary data at *ECCO-JCC* online.

HC, healthy controls; CD, Crohn’s disease; UC, ulcerative colitis; IBD, inflammatory bowel disease; CI, confidence interval.

### 3.2. Trm are deficient in Crohn’s disease and ulcerative colitis

Using this analysis, we next compared numbers and phenotype of CD8^+^ and CD4^+^ Trm and γδ T cells obtained from right colon of HC, CD, and UC donors [Figure 2]. We found a dramatic decrease in both CD8 Trm and γδ T cells in IEL in IBD patients [Figure 2a; 84% and 61% for CD8 Trm; 90% and 87% for γδ T cells in CD and UC, respectively]. Both total numbers and percentages of Trm relative to total live cells were reduced in IBD donors. The phenotype of CD8 Trm was unchanged, but significantly decreased γδ T cell expression of CD39 was seen in UC and CD, suggesting impaired regulatory function. In LPL, there were also fewer Trm, although the deficiency was less dramatic than in IEL [61% and 44% for CD8; 28% and 68% for CD4 in CD and UC, respectively] and did not reach statistical significance for CD4 Trm in CD or when expressed in percentage terms [Figure 2b]. There was no change in phenotype of LPL Trm in disease. Total yields of viable cells in IEL and LPL were unchanged in IBD [Figure 2c]; thus deficiencies in Trm were selective and could not be explained by loss of epithelium. We also found a strong correlation between numbers of CD8 Trm in IEL and γδ T cells [Figure 2d], suggesting co-dependence of these populations. In left colon biopsies we found significantly fewer Trm [Supplementary File 2 and Figure S1, available as Supplementary data at *ECCO-JCC* online] and no significant changes in IBD. The levels of Trm and γδ T cell deficiency were not related to disease severity, as assessed by macroscopic inflammation, in either disease [Figure S2, available as Supplementary data at *ECCO-JCC* online ].

### 3.3. Human Trm development in vitro is controlled by TGF-β, IFN-β, retinoic acid, and AhR receptor agonists

**Figure 3.**
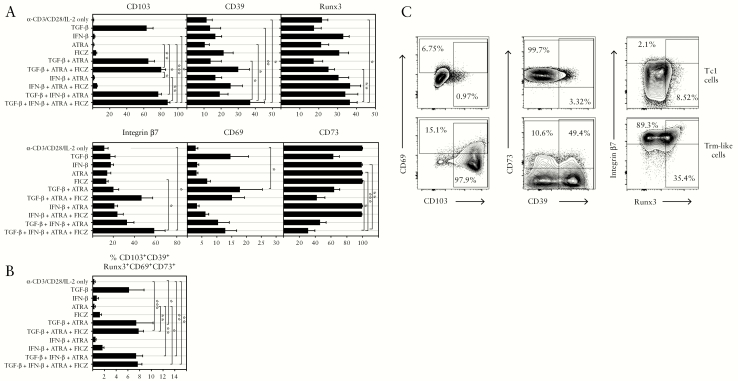
Human CD8 Trm development in vitro is regulated by cytokines, vitamins, and dietary factors. A: Effects of combinations of TGF-β, IFN-β, all-trans retinoic acid [ATRA], and an AhR agonist [FICZ] on Trm, Treg, and homing markers in CD8 effector cells derived from CD8 naïve T cells differentiated with anti-CD3/CD28+IL-2 for 7 days. Graphs show mean ± standard error of the mean [SEM] from five independent experiments; groups compared using one-way ANOVA with Dunn’s test for multiple comparisons applied. B: Cells expressing all Trm-associated markers simultaneously were analysed as in A. C: Staining profiles as in A, showing example of cells cultured in anti-CD3/28 + IL-2 only [Tc1 cells] or with addition of TGF-β, IFN-β, ATRA and FICZ [Trm-like cells].

To determine possible mechanisms contributing to the deficiency of Trm in IBD, we studied micro-environmental factors. We developed an in vitro model to induce human Trm-like cells from naïve CD8 T cells purified from healthy donor PBMC [Figure 3]. Seven days’ differentiation with anti-CD3/CD28 and IL-2 yielded effector cells with few markers of Trm with the exception of the CD73 Treg-associated molecule, and were designated Tc1 type. We tested addition of: TGF-β, a mucosal cytokine known to promote mouse Trm development^2^; IFN-β, since type 1 interferon in the gut can control colitis^13^; all-trans retinoic acid [ATRA], known to induce CD103^14^; and FICZ [5,11-dihydroindolo[3,2-*b*]carbazole-6-carboxaldehyde], an aryl hydrocarbon receptor [AhR] agonist known to promote development of IEL.^15^ Different Trm markers were induced differentially by each factor or combinations thereof. CD103 expression was dependent on TGF-β alone; CD39 was induced by IFN-β and FICZ, as was the Trm transcription factor Runx3. Integrin-β7, not a Trm marker but indicative of gut homing potential, was induced by a combination of TGF-β and ATRA, as was CD69, a Trm marker expressed on all intestinal T cells. Cells expressing all Trm-associated markers simultaneously [Figure 3b] were maximal using a combination of all four factors, which were therefore used to induce Trm-like cells in further functional experiments. IL-15, although involved in mouse Trm development, had no effect in this model.

### 3.4. B cells are dysregulated in quiescent IBD patients

**Figure 4.**
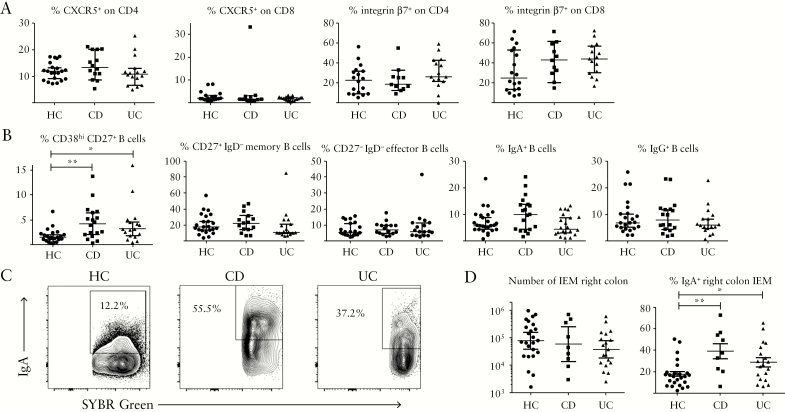
Immunopathology of quiescent IBD reflects B cell dysregulation. A: Proportions of Tfh-like [CXCR5^+^] CD4/CD8 T cells and gut-homing [integrin-β7^+^] T cells in PBMC of healthy control and IBD donors. B: Circulating plasmablasts [CD38^hi^ CD27^+^ CD19^+^] and B cell subsets in healthy and IBD donors. Kruskal-Wallis tests were used to compare groups [*n* = 23 HC; *n* = 18 CD; *n* = 17 UC]. C: IgA coating of IEM obtained from right colon biopsies of example HC, CD, and UC donors, after gating on SYBR Green^+^ events. D: Pooled data showing proportions of IgA^+^ IEM in donor groups. One-way ANOVA was used to compare groups [*n* = 25 HC; *n* = 9 CD; *n* = 19 UC]. IBD, inflammatory bowel disease; PBMC, peripheral blood mononuclear cells; HC, healthy controls; CD, Crohn’s disease; UC, ulcerative colitis; IEM, intraepithelial microbes.

To determine if reduced CD8 T cells in colonic tissue of IBD patients was indicative of imbalance in cell-mediated immunity versus humoral immunity towards the microbiota, we examined proportions of follicular helper [Tfh-like] cells, key inducers of antibody production through interaction with B cells in germinal centres, alongside the gut-homing function of T cells in PBMC [Figure 4a and Supplementary Figure S3, available as Supplementary data at *ECCO-JCC* online]. Tfh-like cells expressing CD4 and CXCR5 were unchanged in IBD, as were proportions of integrin-β7^+^, gut-homing cells. CD8 T cells did not express CXCR5 but showed high levels of integrin-β7 indicating that their gut-homing capacity was not impaired in IBD. Analysis of B cell subsets in PBMC [Figure 4b, Table 2] showed significantly increased proportions of plasmablasts [CD38^hi^ CD27^+^ B cells] in both CD and UC; these are a highly activated subset destined to become plasma cells in tissues.^16^ Other B cell subsets, including those switched to IgA or IgG production, were unchanged. Consistent with increased B cell activity, IEM released from colonic biopsies showed significantly higher levels of IgA coating in both CD and UC than in HC [Figure 4c and d], although numbers of microbes obtained were unchanged.

### 3.5. T and B cell memory responses to commensal bacteria indicate skewing to humoral immunity in IBD

**Figure 5.**
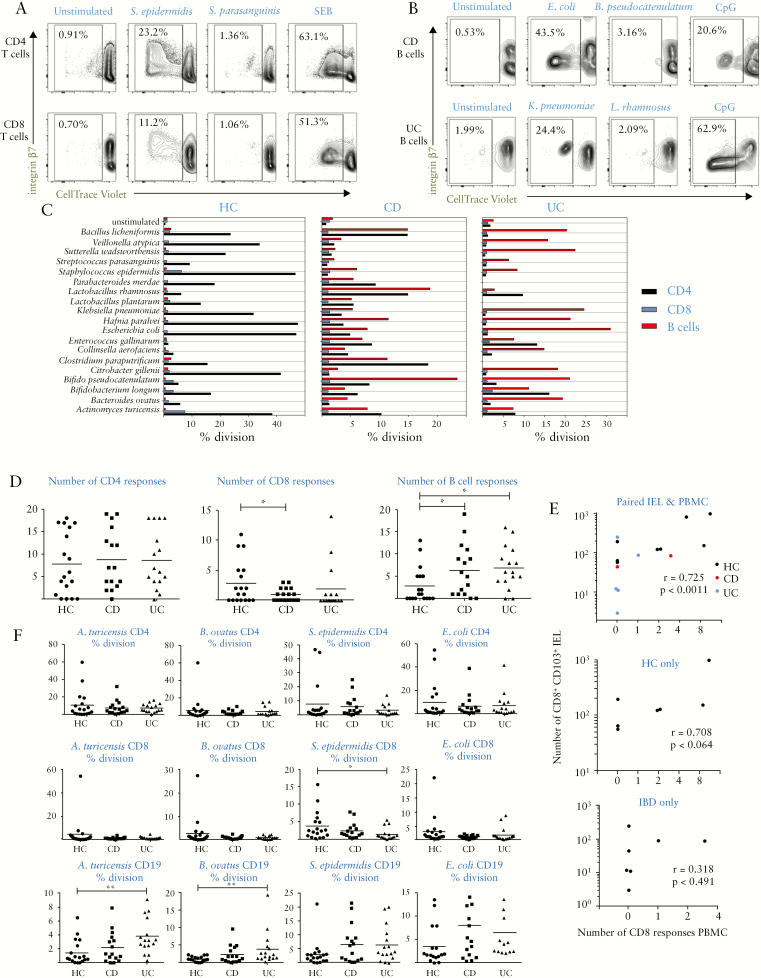
T- and B-cell memory responses to commensal bacteria show skewing from cell-mediated to humoral immunity in IBD. A: CD4 and CD8 T cell memory responses to selected commensals in healthy PBMC, showing examples of CellTrace Violet dilution in CD4/CD8-gated populations in cultures showing positive and negative responses alongside SEB positive control; integrin-β7 staining indicates gut-homing potential of expanded antigen-specific cells. B: CD19^+^ B cell responses to selected commensals in example CD and UC patient PBMC; as in A but gated on CD19^+^ events. C: Representative proliferation data in PBMC from an HC, CD, and UC donor, showing responses to a panel of 19 bacteria after 7 days stimulation and gating for CD4^+^ CD8^+^ and CD19^+^ cells. D: Pooled data as in C, showing numbers of positive responses within panel of 19 commensals. Kruskal-Wallis tests were used to compare groups; *n* = 18 HC, *n* = 16 CD and UC. E: Correlation of CD8 proliferative responses in PBMC with CD8 Trm in IEL from autologous biopsies [*n* = 15]. Upper panel shows pooled data with lower panels showing individual correlations for HC and IBD samples. F: Magnitude of proliferative responses to 4 individual species, one from each phylum. Pearson correlation coefficient was calculated in E. IBD,inflammatory bowel disease; PBMC, peripheral blood mononuclear cells; HC, healthy controls; CD, Crohn’s disease; UC, ulcerative colitis; IEL, intraepithelial lymphocytes.

We then analysed whether antigen-specific T and B cell responses to specific commensal bacteria were imbalanced in IBD [Figure 5]. We selected 19 commensal strains mainly isolated from healthy human caecum, covering as many genera as possible. Killed bacteria were added to PBMC for 7 days to identify specific memory CD4/CD8 T cell or B cell proliferative responses. Results showed responses were highly specific to individual species [Figure 5a and b] and showed high degrees of variability both between individual donors and between HC and IBD patients [Figure 5c]. Variability within responses of individual donors was noted after around 1 year [Supplementary Figure S4, available as Supplementary data at *ECCO-JCC* online], indicating that such memory is dynamic and not long-term. As expected, CD4 T cell responses were the predominant memory response in all groups; however, total numbers of positive responses for each donor were unchanged in health vs IBD [Figure 5d]. By contrast, numbers of the less frequent CD8 T cell responses were significantly reduced in CD compared with HC, with the same trend apparent in UC [Figure 5d]. B cell memory responses to bacteria were rare in health but significantly increased in both CD and UC [Figure 5d]. T cells proliferating in response to microbes expressed integrin-β7 [gut-homing marker], CLA [skin-homing], and the CD39 Treg marker, whereas B cells only expressed CLA in response to microbes [Figure 5a; Figure S3].

**Figure 6.**
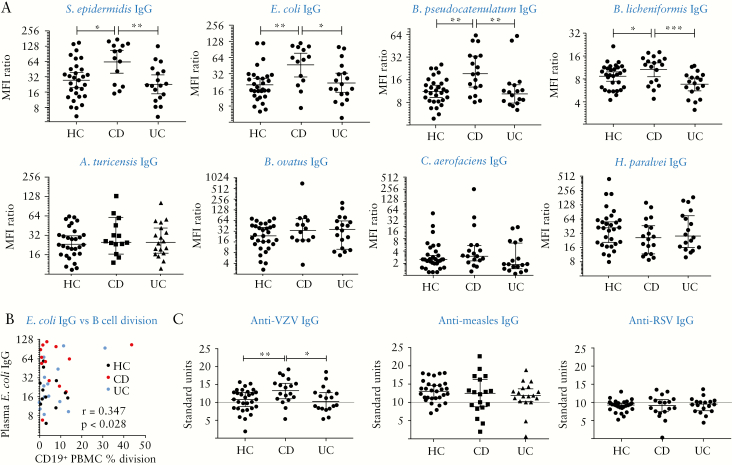
Circulating specific IgG antibodies to immunogenic commensal species are raised in CD but not UC donors. A: Plasma was assayed for IgG antibodies using a coating assay. Ratios of median fluorescence intensity of anti-IgG-stained vs isotype control for each sample are shown [median and 95% CIs]. Kruskal–Wallis tests were used to compare groups; *n* = 30 HC, *n* = 18 CD and UC. B: Correlation of B cell proliferative responses to *E. coli* against circulating IgG in 40 matched donors, including Pearson correlation coefficient. Correlations with other species were not significant. C: Antibodies against non-enteric viruses in health vs IBD. Plasma were assayed by ELISA for IgG to viral antigens and results expressed in arbitrary units. Grey lines represent cut-off points below which titres are considered negative. One-way ANOVA was used to compare groups. IBD,inflammatory bowel disease; HC, healthy controls; CD, Crohn’s disease; UC, ulcerative colitis; CI, confidence interval.

To investigate a possible link between circulating CD8 memory to commensals and recruitment of CD8 Trm to mucosa, we correlated numbers of CD8 responses to the 19 bacteria with CD8 Trm [IEL] numbers in donors where both blood and biopsies were obtained; this indicated a significant positive correlation [Figure 5e]. We then examined the magnitude of individual responses, as reflected by the proportion of divided cells, which is related to antigen-specific precursor frequency. Results for the most immunogenic species from each phylum [Figure 5f] show the high level of variability between donors, with a significant difference in CD8 response in health vs IBD revealed for *Staphylococcus epidermidis* only, the most immunogenic species. B cell responses, however, were significantly increased for all species in CD and UC. We also categorised numbers of proliferative responses against the four phyla of bacterial species [Figure S5, available as Supplementary data at *ECCO-JCC* online]. The same trends were observed in all phyla, with the most significant differences in CD8 and B cell responses seen in Actinobacteria and Firmicutes. We also performed assays for commensal-specific antibodies in plasma, a more conventional readout for B cell immunity. Circulating IgG specific for the most immunogenic species in the 19-strain panel, as shown in Figure 6a, showed that antibody was increased in CD but not UC-indeed, levels in UC were the same as those in HC and significantly lower than in CD. Antibodies to less immunogenic species were detectable but not significantly different between health and IBD. The divergent findings with B cell proliferative response vs circulating IgG were reflected in poor correlations between the levels of each in individual donors; the only statistically significant correlation was found with *E. coli* [Figure 6b]. Circulating IgA levels were much lower than IgG [Supplementary Figure S6,] available as Supplementary data at *ECCO-JCC* online or undetectable, and did not show significant differences between health and disease.

We tested whether immune deviation seen in IBD was specific to microbiota or reflected a systemic bias affecting responses to other antigens. We chose to assess responses to classic CD8 T cell-inducing viral antigens. Plasma were assayed for IgG to three non-enteric viruses encountered in childhood—varicella zoster [VZV], measles, and respiratory syncytial virus [RSV] [Figure 6c]. Antibody to VZV showed the same pattern as commensals, with significantly increased levels in CD but not UC. Antibodies to measles were detectable in all patient groups but did not differ significantly, whereas few positive titres of RSV IgG were detected.

### 3.6. Mechanisms of immune deviation in IBD

**Figure 7.**
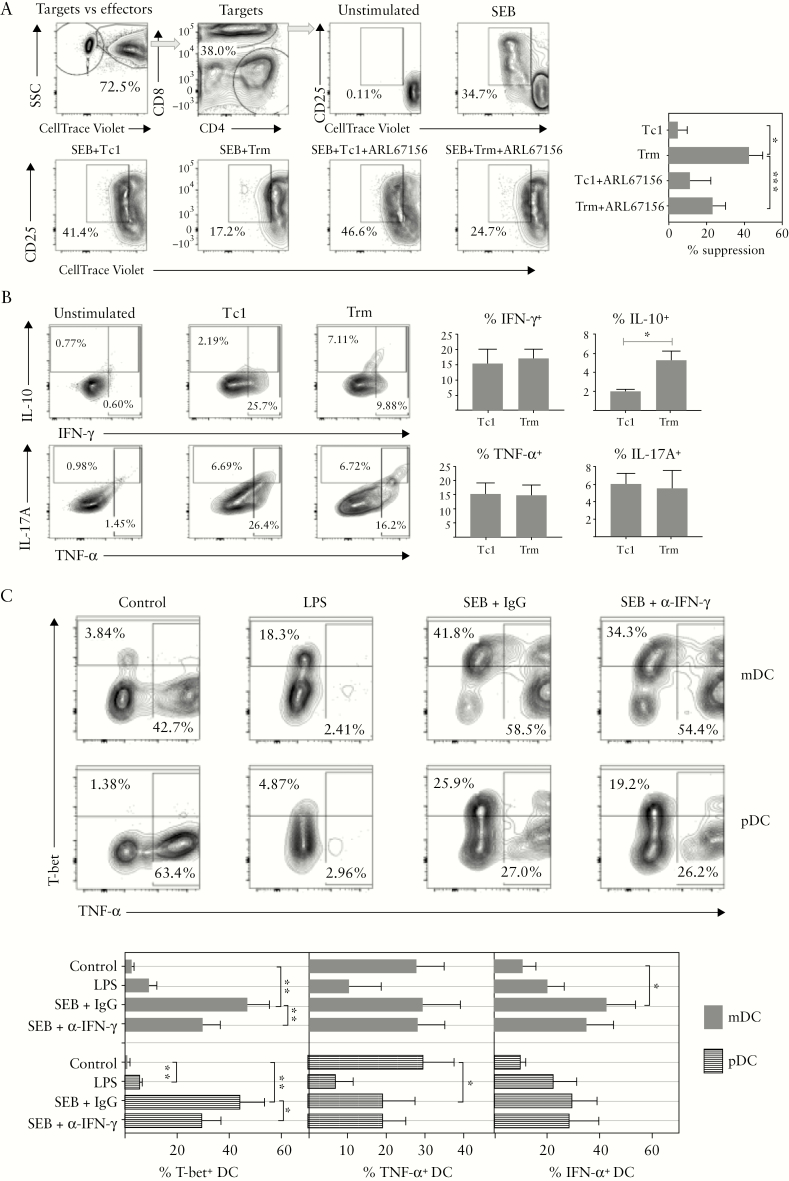
Mechanisms of immune deviation in IBD. A: Trm-like cells have Treg function partially dependent on CD39 nucleotidase activity - Tc1 or Trm-like cells were added to autologous fluorescent labelled PBMC and suppression of CD8 target cell activation was determined in the presence or absence of the CD39 inhibitor ARL67156. Example staining and pooled data showing % suppression of proliferation from three independent experiments – mean ± SEM, paired t-tests used to compare groups. B: Trm-like cells have increased capacity for IL-10 secretion. Tc1 and Trm-like cells were restimulated with PMA/ionomycin/monensin and stained for intracellular cytokines. Example staining and pooled data showing % staining from four independent experiments - mean ± SEM, paired t-tests used. C: Induction of T-bet expression in DC by cognate interaction with T cells mediated by superantigen is associated with altered cytokine synthesis; PBMC were cultured overnight with LPS or SEB plus control antibody/anti-IFN-γ, followed by 4 h with LPS+poly I:C+monensin. mDC/pDC populations were gated according to CD11c/CD123 expression after gating on singlet, viable DC using lineage vs HLA-DR plots. Example staining and pooled data from four to five independent experiments are shown; paired t-tests were used to compare groups. IBD,inflammatory bowel disease; PBMC, peripheral blood mononuclear cells; HC, healthy controls; CD, Crohn’s disease; UC, ulcerative colitis; SEM, standard error of the mean.

The above data clearly indicated a pattern of immune deviation between cellular/cytotoxic and humoral immunity to members of the intestinal microbiota as an underlying feature of IBD. To examine potential pathogenic mechanisms, we first pursued the hypothesis that Trm express regulatory T cell function. We performed suppression assays using conventional Tc1-type CD8 effector cells and Trm-like cells, generated using our in vitro model system [Figure 3]. The targets used in the assays were autologous PBMC stored in liquid nitrogen. Effector cells were added at a 1:4 ratio and an inhibitor of CD39 ectonucleotidase activity [ARL67156] was used to determine whether suppressive activity was CD39-dependent. Target CD8 T cell proliferation assayed after 4 days revealed suppressive activity in Trm but not in Tc1 cells, which was partially reversed in the presence of the CD39 inhibitor [Figure 7a]. The cultured Tc1 and Trm cells were also tested for cytokine production [Figure 7b], which revealed that Trm cells had similar capacity for production of pro-inflammatory cytokines IFN-γ, TNF-α, and IL-17 compared with Tc1; however IL-10, the key immunoregulatory cytokine in the GI tract,^17^ was significantly increased in Trm cells. Since dendritic cells [DCs] are critical for controlling immune deviation and tolerogenic responses and are a target of Treg, we analysed the interaction between T cells and DCs, again using a model system with healthy PBMC. To stimulate DC: T cell cognate interactions we used SEB superantigen, compared with TLR-mediated DC stimulation using LPS [Figure 7c]. We also added LPS + poly I:C + monensin to cultures for the final 4 h, in order to assess DC cytokine production. SEB but not LPS strongly induced T-bet transcription factor expression in both myeloid [mDC] and plasmacytoid DC [pDC], and those DC expressing T-bet produced less TNF-α and more IFN-α, cytokines with opposing roles in colitis.^13,18–20^ In pDC, overall TNF-α production was suppressed by SEB, whereas in mDC the reduced levels in T-bet^+^ DC were counterbalanced by increased TNF-α in T-bet– cells. The effect of T cell:DC interaction on T-bet was partially dependent on IFN-γ, as shown by a neutralising IFN-γ antibody, but effects on cytokine production appeared IFN-γ independent.

## 4. Discussion

IBD is characterised by acute inflammatory episodes and pathology, and current treatments aim to suppress symptoms using a plethora of immunosuppressive strategies. Our studies here, focusing mainly on patients with no ulceration [CD] or a Mayo index of 1 or less [UC], reveal that underlying disease is characterised by reduced CD8 T cell immunity to commensal microbes associated with a paucity of Trm, potentially explaining the loss of barrier immunity which characterises IBD and drives pathology. Reduced CD8 response can also explain the skewing of immunity towards B cell-mediated antibody production, and loss of immunoregulation in the local mucosa due to the reduced numbers of cells expressing key Treg molecules CD39 and CD73. The mutually antagonistic relationship between cell-mediated and humoral immunity was first noted in the 1970s^21^ and was subsequently attributed to the Th1/Th2 axis,^22,23^ as was the hygiene hypothesis in immune-mediated disease.^24^ CD8 T cell responses skew immunity away from humoral and towards cellular immunity—our study is the first to examine such responses to the intestinal microbiota in humans and points towards novel strategies in IBD treatment. Studies of anti-inflammatory commensal-induced pathways in the gut have focused on CD4 Foxp3 Treg, which form a small fraction of the LPL and are absent from IEL in human colon. By contrast, our data show Trm could provide a gatekeeper function, controlling access of mucosal antigens to germinal centres in lymphoid tissue, and thus Tfh:B cell interaction, while simultaneously controlling inflammation through breakdown of extracellular ATP.^25,26^ Runx3 has recently been defined as a master transcription factor for development of murine Trm.^11^ Our data show that human gut Trm preferentially express Runx3, and further co-express CD39 and CD73, key functional molecules on Treg cells.^27^ CD39 is essential for in vitro suppressive activity of Foxp3^+^ Treg cells due to its ability to degrade extracellular ATP,^28^ which activates DC,^29^ and CD73 assists further nucleotide breakdown to adenosine, an immunosuppressive molecule.^30^ ATP is released in mucosal tissue by injury but is also secreted by bacteria,^31^ explaining the necessity for high expression of these molecules by Trm, especially IEL, in comparison with circulating T cells. IEL and in vitro-derived Trm-like cells expressed lower levels of CD73, suggesting that further breakdown of ADP towards adenosine occurs further into the mucosa. It should be noted that there are multiple other mechanisms via which Treg may express their function,^32^ and we have not been able to directly assess suppressive activity in tissue-derived Trm or compare such activity in health vs IBD, due to the low numbers available. Therefore, unchanged levels of CD39 and CD73 on Trm in IBD does not exclude the possibility of altered function.

Foxp3^+^ Treg are critical in systemic tolerance and in establishing tolerance to self-antigens in early life.^33^ Foxp3 was not expressed in CD8 T cells in the colon, which outnumber CD4 T cells. Foxp3^+^ Treg were present at a modest percentage in the CD4 LPL population [around 5%] and were vastly outnumbered by Foxp3-negative CD4 and CD8 T cells, mostly Trm, expressing high levels of CD39 and CD73. Arguably the low number of Foxp3^+^ Treg in human colon is insufficient to maintain tolerance in the presence of such large antigenic loads from the microbiota, necessitating accumulation of Trm populations with regulatory capacity. Since Trm do not differentiate until they reach the tissue,^34^ this would explain why we found tolerance to commensal bacteria was not systemic, but localised to the gut. Circulating T cells reactive to commensals would not express regulatory function until resident in the tissue, and would require local tissue factors such as type I IFN and AhR agonists to maintain their function. This picture contrasts with that emerging from mouse models, most likely due to far greater antigenic experience and maturity of the adult human immune system compared with laboratory mice. We found higher proportions of conventional CD8αβ T cells in tissue than reported in mice, which may rely more on innate mechanisms and thymus-derived Foxp3^+^ Treg, due to their short lifespan. We also found strong correlation between αβ ^+^ and γδ ^+^ T cells in IEL, implying that αβ ^+^ may support γδ ^+^ cell populations in a fashion analogous to that demonstrated in the thymus,^35^ or co-dependence on tissue-specific environmental factors. A further correlation was shown between numbers of memory CD8 responses to commensal bacteria and colonic CD8 Trm, suggesting that such responses are required to recruit and maintain healthy Trm populations. CD8 Trm have recently been shown to be recruited to skin in response to skin resident microbes in a non-classical major histocompatibility complex [MHC]-restricted fashion.^36^ This mouse study demonstrated that such Trm exhibited an unusual phenotype with expression of immunoregulatory genes and wound-healing activity, thus improving barrier function without inflammation. Our data suggest that a similar phenomenon occurs in the colon but is dependent on classical responses to a wide range of bacterial antigens.

One recent study demonstrated a pro-inflammatory role for Trm cells in active IBD,^37^ thus suggesting that Trm can exhibit both pro- and anti-inflammatory activities dependent on the context. CD4 and CD8 Trm were increased in the lamina propria of inflamed IBD tissue in this study, and T cell transfer colitis experiments in mice confirmed that T cells adopt a Trm phenotype soon after recruitment to lamina propria in active disease.^37^ The pathological role of Trm was dependent on their pro-inflammatory cytokine production regulated by Hobit/Blimp-1 transcription factors expressed in Trm. However, deletion of Hobit/Blimp-1 in mouse CD4 T cells had no effect on their regulatory function or development into Trm.^37^ A further study compared proportions of CD103^+^ cells within total T cell populations in inflamed vs uninflamed biopsies from CD and UC patients.^38^ This study showed decreased proportions of CD103^+^ cells in inflamed tissue; however, this could have been due to influx of effector memory-type cells. Further work in murine systems targeting regulatory function in Trm and effects on disease susceptibility are therefore warranted. We propose that dual functionality of Trm cells in homeostatic versus inflammatory conditions would allow balanced immunity to occur across large areas of tissue exposed to high antigenic loads.

Murine studies have not clearly described the biology of CD4^+^ Trm and, unlike CD8 cells, Runx3 expression in human CD103^+^ CD4 cells in LPL was low, so we focused on CD8^+^ Trm activities. Dietary factors retinoic acid and AhR agonists could play a role in expression of the Trm phenotype within tissue or in Trm survival, in addition to the mucosal cytokine TGF-β and type 1 interferon, both cytokines associated with suppression of colitis.^13,39–41^ Gut type 1 interferon production could be influenced by the enteric virome, which is also altered in IBD.^42^ ATRA is derived from vitamin A by antigen-presenting cells,^39^ whereas AhR agonists are dietary factors contained in cruciferous vegetables and key to gut health.^15^ Combining these factors in vitro allowed us to develop the first in vitro differentiation model for human Trm, but also revealed distinct regulation of individual Trm-associated markers. Interestingly, although CD103 expression was entirely dependent on TGF-β, the key Trm transcription factor Runx3, and CD39 were unaffected by TGF-β and co-regulated by type 1 interferon and the AhR agonist. Thus CD103, as a migration marker, may not necessarily reflect Trm function in clinical studies.

Circulating IgG antibody to certain commensals was increased in CD but not UC, despite B cell dysfunction in UC with increased circulating plasmablasts and IgA secretion. Since inflammatory lesions penetrate deeper into intestinal tissue in CD than in UC,^43^ and there is more involvement of mesenteric lymph nodes in CD than in UC,^44,45^ it is possible that longer-lived, higher-affinity antibody responses are generated in CD, as antigens could access germinal centres in lymph nodes driving affinity maturation. Circulating B cell proliferative responses may reflect shorter-term responses with more broadly reactive antibody synthesis focused on mucosa. Indeed, repeat assays on individual healthy donors showed memory responses to commensal bacteria could change within a year, and long-term memory is not required for non-pathogenic organisms. The excessive IgA response to mucosa-associated microbes was apparent in both CD and UC but was not accompanied by increased circulating Tfh-like cells. Future work could examine microbe-specific Tfh cells, but these might be sequestered in lymphoid tissue.

Mechanisms through which CD8 T cells might control immune deviation are not entirely clear, but they are known to regulate CD4 T cell development. Here CD4 responses were not altered in IBD, but we did not examine their cytokine profiles, which are skewed towards a Th17 profile in IBD.^4^ Th17 development is strongly inhibited by IFN-γ,^46^ the major product of CD8 T cells. Our data show that an additional mechanism could be via induction of T-bet in DCs, either via interaction with tissue-resident T cells reactive to microbial antigens, or in draining lymphoid tissues. T-bet expression in DC is critical in preventing colitis in mice, since it represses production of TNF-α.^18,47^ Consistent with this concept, we found interaction with T cells suppressed TNF-α in pDC while enhancing IFN-α production in DC, although these effects were less specific to T cells and IFN-γ. This novel pathway may contribute to immune deviation and allow acquired immune memory to reinforce DC activity in tissues. Dialogue between Trm and DC in tissue may inform appropriate type of memory response as well as directing tissue migration of effector cells. Current dogma states that DC direct T cell responses after integrating signals from innate immunity and tissue damage. However, additional dialogue between tissue DC and Trm would allow for more intelligent decision making based on host immunological experiences, thus allowing the gut immune system to learn which bacteria are pathogenic over time.

IBD is a clear example of a ‘Western’ disease associated with dysbiosis and disrupted immunoregulation.^48^ Our data establish IBD as a disease of B cell dysfunction and point towards deficient CD8 T cell priming to the microbiota as key to its aetiology. IBD often exhibits extra-intestinal manifestations,^49^ and several other diseases are associated with intestinal dysbiosis. It is therefore possible that reduced Trm priming is a general mechanism underlying the hygiene hypothesis in immune-mediated disease, and associations of microbiota with tumour development and cancer therapy.^50,51^ Memory CD8 T cells migrate to multiple tissues and escape homeostatic control mechanisms that limit their numbers in the circulation,^52^ so numbers of Trm can accumulate throughout life in response to immunological experiences. Notably, CD8 Trm accumulate throughout childhood in humans,^53^ when IBD is often first diagnosed. Since CD8 responses are typically used for dealing with highly pathogenic organisms, a lack of exposure to enteric pathogens in early life could result in weakened tissue immunity and thus an altered microbiota. Evidence for this in IBD was provided by the increased antibody response to VZV seen in CD. VZV is latent and requires constant immune surveillance by cytotoxic T cells; thus increased antibody may reflect weaker cytotoxic control of virus, although this was not the case for measles and RSV.

Manipulating immunity to intestinal microbiota through vaccination may address the underlying disease process, unlike current immunosuppressive strategies. It may prove of greater therapeutic benefit than changing the microbiota itself in a range of diseases associated with dysbiosis, since every patient will respond differently to any particular microbe/cohort, due to MHC differences. Mice that lack T-bet expression in their innate immune system develop altered microbiota which is colitogenic,^18^ indicating that dysbiosis is secondary to immune changes. Vaccination would need specifically to target CD8 T cell responses; inducing cytotoxic activity against target microbes may eliminate them from the microbiota, thus preventing pathology. The concept that immunisation to induce CD8 T cell responses can suppress inflammatory pathology may be counter-intuitive, but proof of principle for this was demonstrated in mouse models of airway disease.^54,55^ Such vaccination could provide long-lasting effects on the highly plastic DCs that direct immune responses into pathways associated with health or disease.

## Supplementary Material

jjz175_suppl_Supplementary_Methods

jjz175_suppl_Supplementary_Figures
