# Supplementary material for: Deficient Resident Memory T Cell and CD8 T Cell Response to Commensals in Inflammatory Bowel Disease
Source: J Crohns Colitis. 2019 Oct 26;14(4):525–37. doi: 10.1093/ecco-jcc/jjz175 (PMC7242004; doi:10.1093/ecco-jcc/jjz175)
Supplement: jjz175_suppl_Supplementary_Methods [file jjz175_suppl_supplementary_methods.docx]

**Supplementary file 1 - Materials and** **Methods**

Supplementary Methods Table. Antibodies used:

| **ANTIGEN** | **FLUOROCHROME** | **mAb CLONE** | **SUPPLIER** |
| --- | --- | --- | --- |
| Axl | Alexa Fluor 488 | 108724 | R&D Systems |
| CD3 | PE-Cy5 | UCHT1 | BD Biosciences |
| CD3 | None | HIT3a | BioLegend |
| CD4 | BB515 | SK3 | BD Biosciences |
| CD4 | APC-Cy7 | OKT4 | BioLegend |
| CD8α | BV605 | SK1 | BioLegend |
| CD8β | PE-Cy7 | SIDI8BEE | eBioscience |
| CD11c | BV605 | B-ly6 | BD Biosciences |
| CD14 | PerCP-Cy5.5 | M5E2 | BD Biosciences |
| CD16 | PE-Cy5 | 3G8 | BD Biosciences |
| CD19 | PE-Cy5 | HIB19 | BD Biosciences |
| CD24 | APC-Cy7 | ML5 | BioLegend |
| CD25 | PE-Cy7 | M-A251 | BD Biosciences |
| CD27 | PE | M-T271 | BD Biosciences |
| CD28 | None | CD28.2 | BioLegend |
| CD34 | PE-Cy5 | 581 | BD Biosciences |
| CD38 | BV605 | HIT2 | BioLegend |
| CD39 | PE-Cy7 | A1 | BioLegend |
| CD45RA | PE-Cy5 | HI100 | BioLegend |
| CD69 | BV605 | FN50 | BioLegend |
| CD73 | APC | AD2 | BioLegend |
| CD103 (integrin αE) | PE | Ber-ACT8 | BioLegend |
| CD123 | PE-Cy7 | 6H6 | eBioscience |
| CXCR5 (CD185) | BV421 | J252D4 | BioLegend |
| CLA | PE | HECA-452 | BioLegend |
| HLA-DR | BV421 | L243 | BioLegend |
| IgA | APC | IS11-8E10 | Miltenyi Biotec |
| IgD | PE-Cy7 | IA6-2 | BD Biosciences |
| IgG | FITC | IS11-3B2.2.3 | Miltenyi Biotec |
| IgM | BV421 | G20-127 | BD Biosciences |
| Integrin β7 | PE | FIB504 | BD Biosciences |
| IFN-α | V450 | 7N4-1 | BD Biosciences |
| IFN-γ | FITC |  | BD Biosciences |
| IFN-γ | None, low endotoxin no azide | B27 | BioLegend |
| IL-10 | APC | JES3-19F1 | BioLegend |
| IL-17A | PE | SCPL1362 | BD Biosciences |
| Runx3 | PE | R3-5G4 | BD Biosciences |
| T-bet | APC | 4B10 | BioLegend |
| TCR γδ | FITC | 11F2 | BD Biosciences |
| TNF-α | PE-Cy7 | MAb11 | eBioscience |


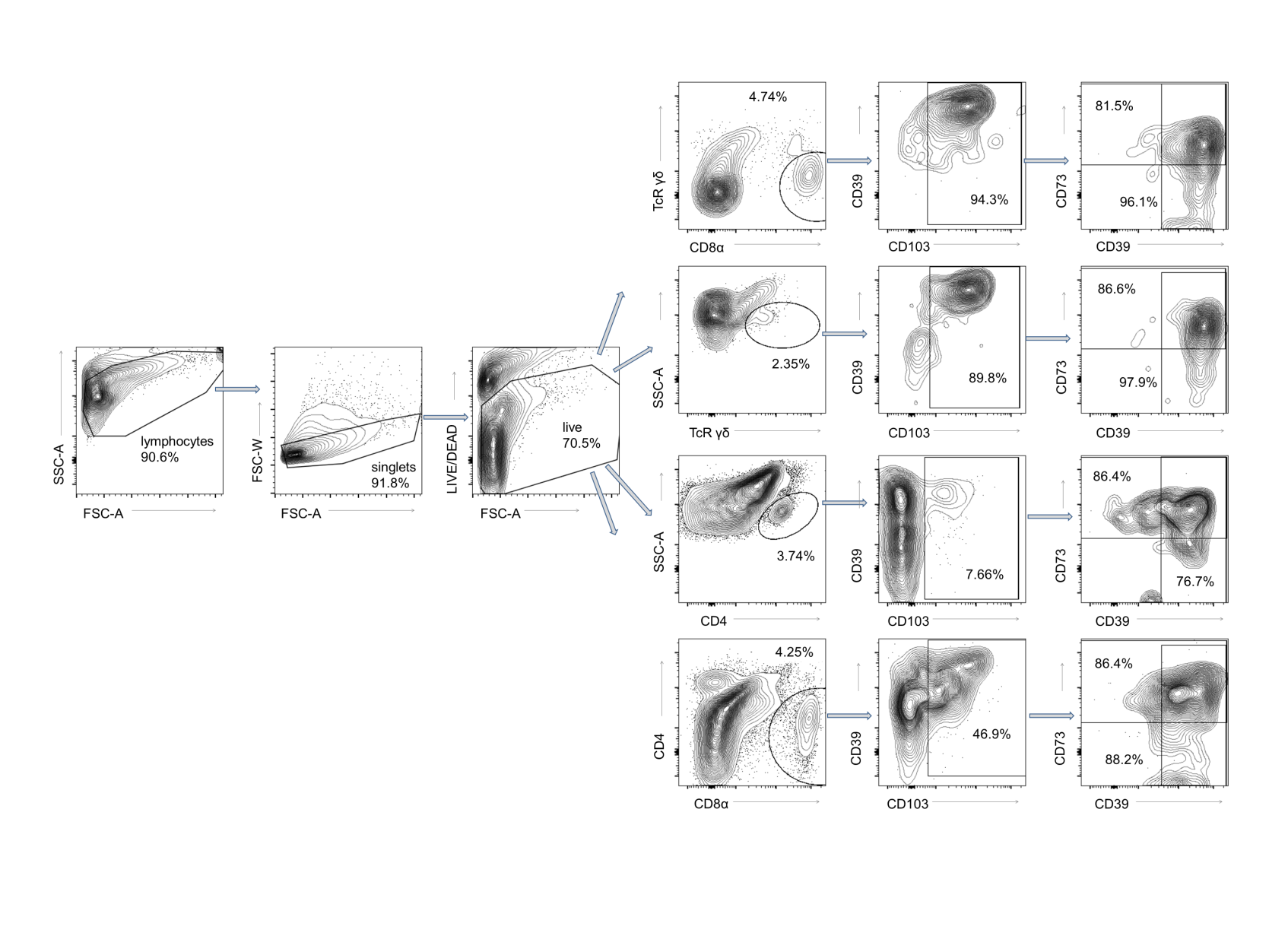


Supplementary Methods Figure 1. Gating strategy for Trm data as in Fig 2. IEL/LPL were sequentially gated for: lymphocytes, single cells and live cells, followed by conventional CD8 T cells and γδ T cells for IEL (top two rows); or CD4 and CD8 T cells for LPL (bottom two rows). CD103^+^ cells within each subset were considered Trm.


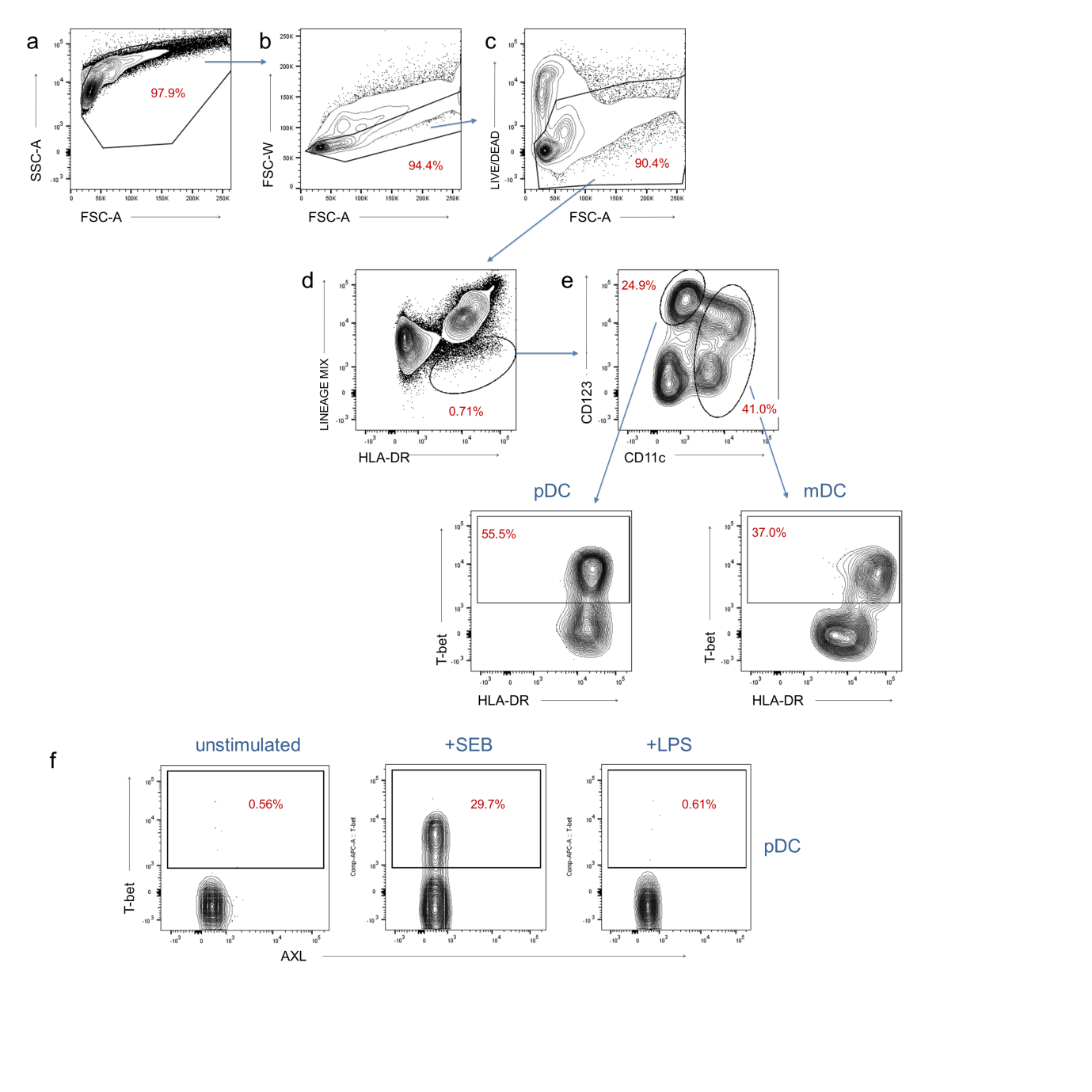


Supplementary Methods Figure 2. Gating strategy for mDC and pDC analysis as in Fig 7c. PBMC were sequentially gated for: lymphocytes (a); single cells (b); live cells (c); total DC (d); pDC and mDC (e). Gated mDC and pDC were analyzed separately for T-bet intranuclear staining. PBMC cultured overnight with SEB are shown. F: pDC-gated cells induced to express T-bet do not express Axl. pDC gated as shown were pre-stained with anti-AXL, a marker indicating contamination with non-pDC. PBMC cultured overnight or stimulated with SEB or LPS are shown.
