# Supplementary material for: Deficient Resident Memory T Cell and CD8 T Cell Response to Commensals in Inflammatory Bowel Disease
Source: J Crohns Colitis. 2019 Oct 26;14(4):525–37. doi: 10.1093/ecco-jcc/jjz175 (PMC7242004; doi:10.1093/ecco-jcc/jjz175)
Supplement: jjz175_suppl_Supplementary_Figures [file jjz175_suppl_supplementary_figures.docx]

**Supplementary file 2 – Figures S1-S6**


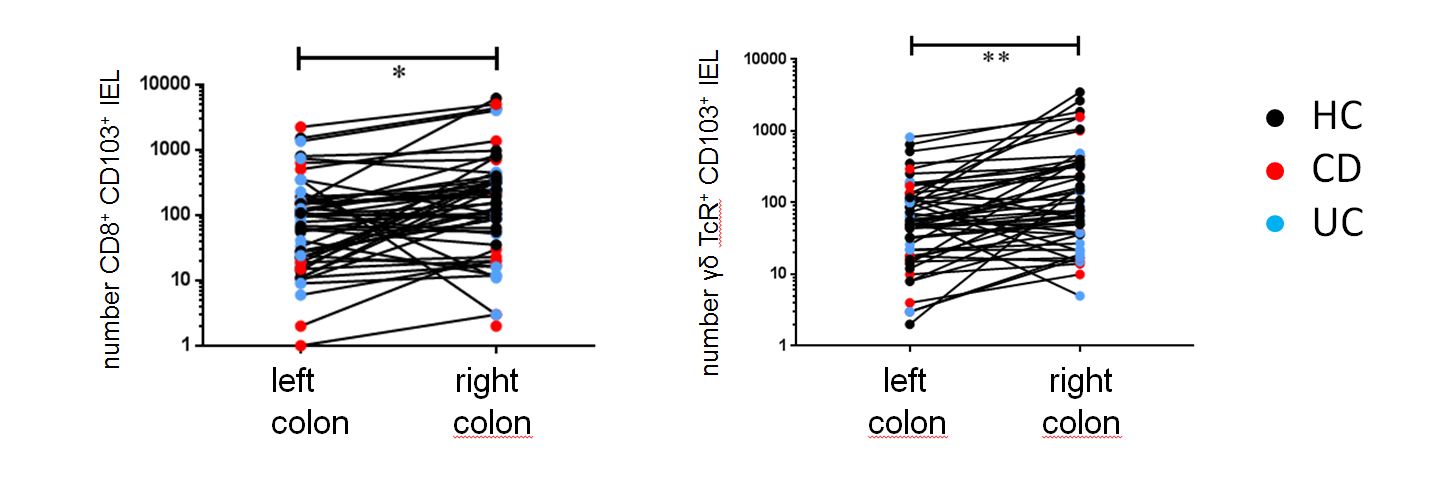


Figure S1. Left colon contains fewer CD8^+^ Trm and γδ T cells than right colon. 5 biopsies were obtained from left and right colon of HC, CD and UC donors and IEL analyzed. Numbers of each cell type extracted are shown, with lines showing differences in individual donors. Paired t tests were used to compare left and right colon data.


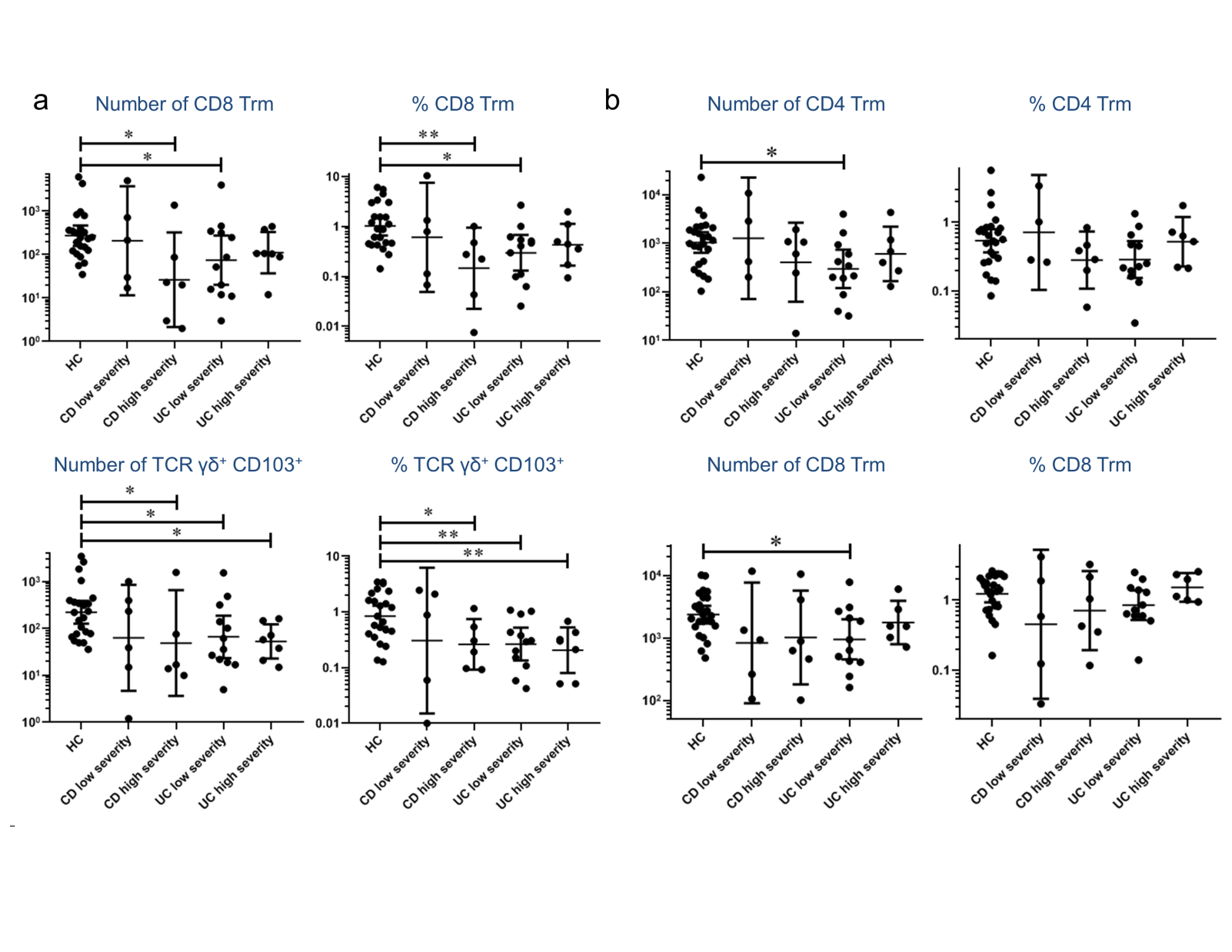
Figure S2. Changes in Trm populations in IBD are not significantly different in low vs high disease severity subgroups. CD patients (as in Figure 2) were divided into low (no ulceration) and high (ulceration) severity groups based on inflammation. UC patients were divided according to endoscopic Mayo scores into low severity (Mayo 0&1) and high severity (Mayo 1&2) groups. A: Numbers and percentages of major IEL populations; B: Numbers and percentages of Trm in LPL. Statistically significant differences between groups are indicated (Kruskall-Wallis test).


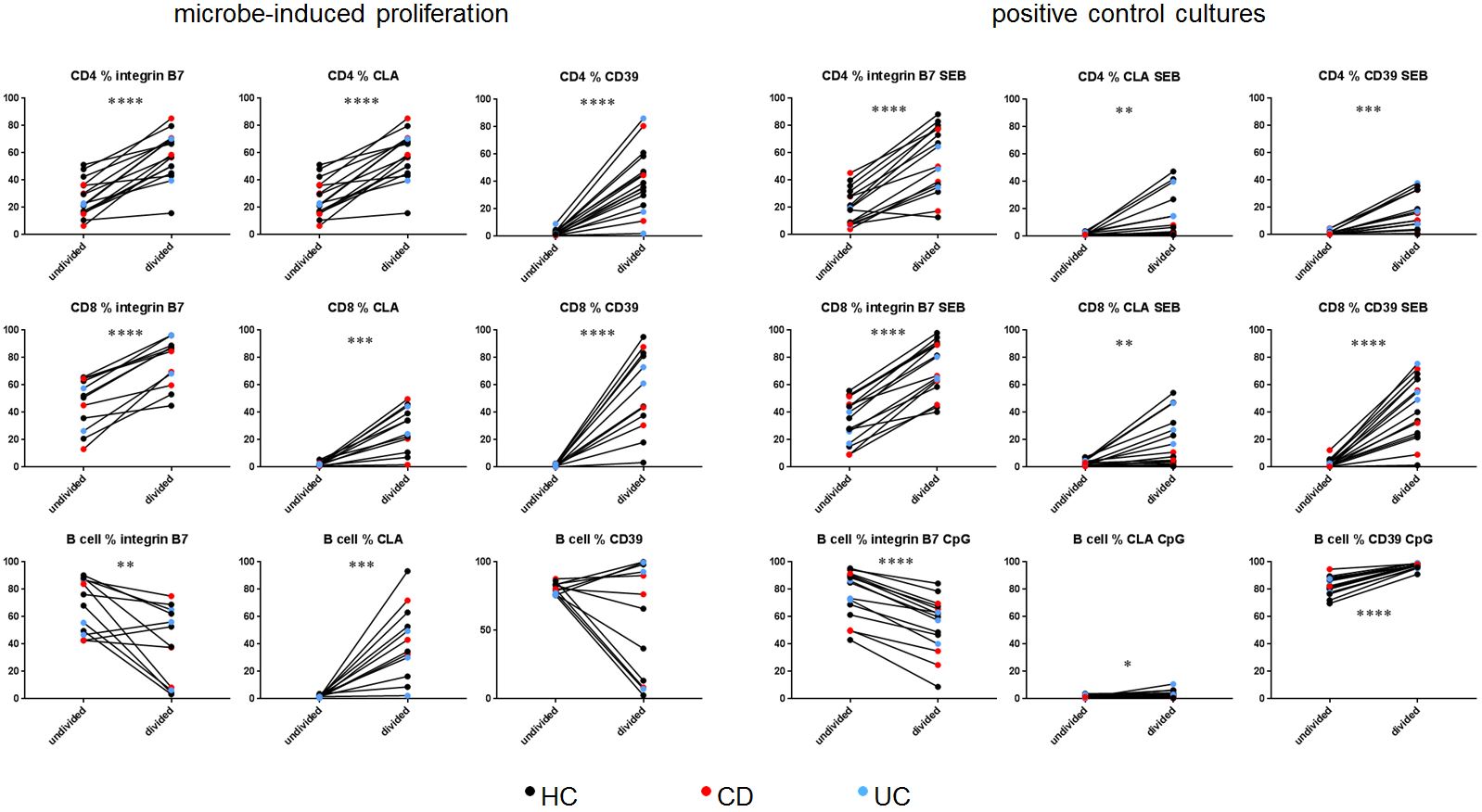


Figure S3. Expression of gut-homing, skin-homing and regulatory markers on lymphocytes responding to commensal microbes (left) or mitogens (right). Lines show differences in integrin β7, CLA and CD39 expression between divided and non-divided cells from the same culture in different donors; experiments were as in Fig 5a. Strongest proliferative responses to each individual microbe, which differed between donors, were selected for each donor (pooled data shown on left), and matching SEB- or CpG-induced T cell and B-cell proliferative responses are shown on the right. Paired t tests were used for statistical analysis (n=12).


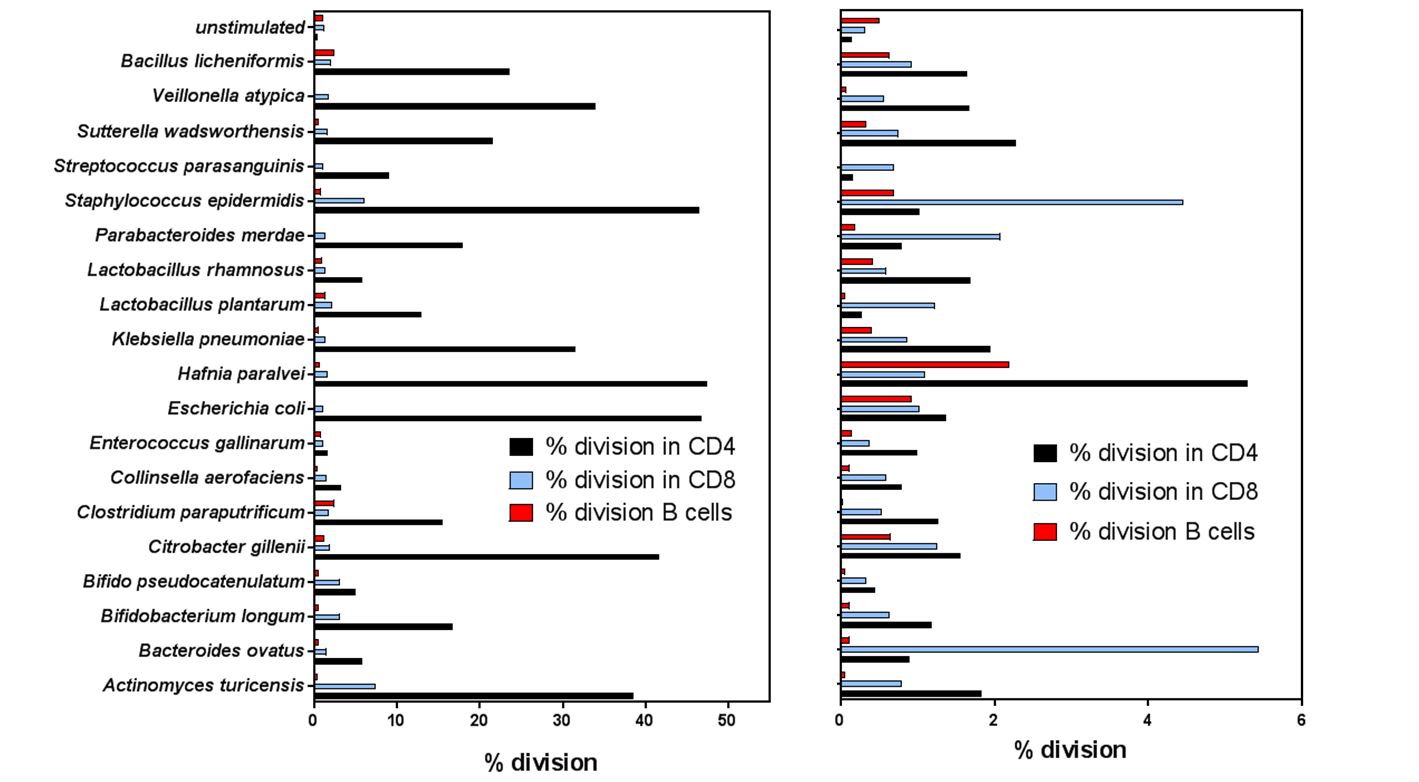


Figure S4. Circulating memory T- and B-cell proliferative responses to a panel of commensal bacteria in a single healthy PBMC donor sampled 11 months apart, showing differences in specificity, magnitude and pattern of response. Similar results were obtained in 4 individuals.


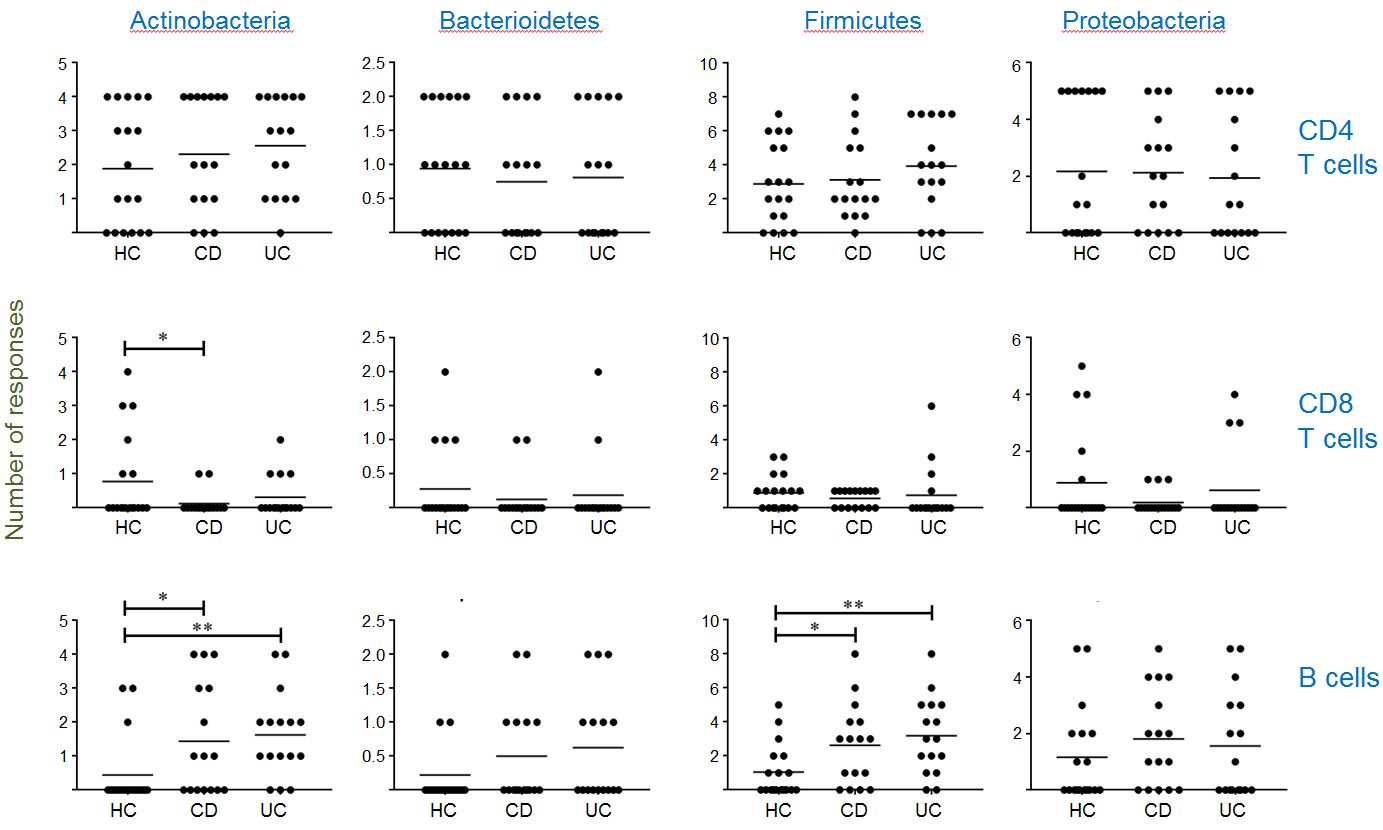


Figure S5. Proliferative responses in PBMC from HC, CD and UC donors, showing responses to a panel of 19 bacteria representing 4 major phyla, after 7 days stimulation and gating for CD4^+^ CD8^+^ and CD19^+^ B cells, as in Figure 5. Numbers of positive responses within each phylum are shown. Kruskal-Wallis tests were used to compare groups (n=18 HC; n=16 CD&UC).


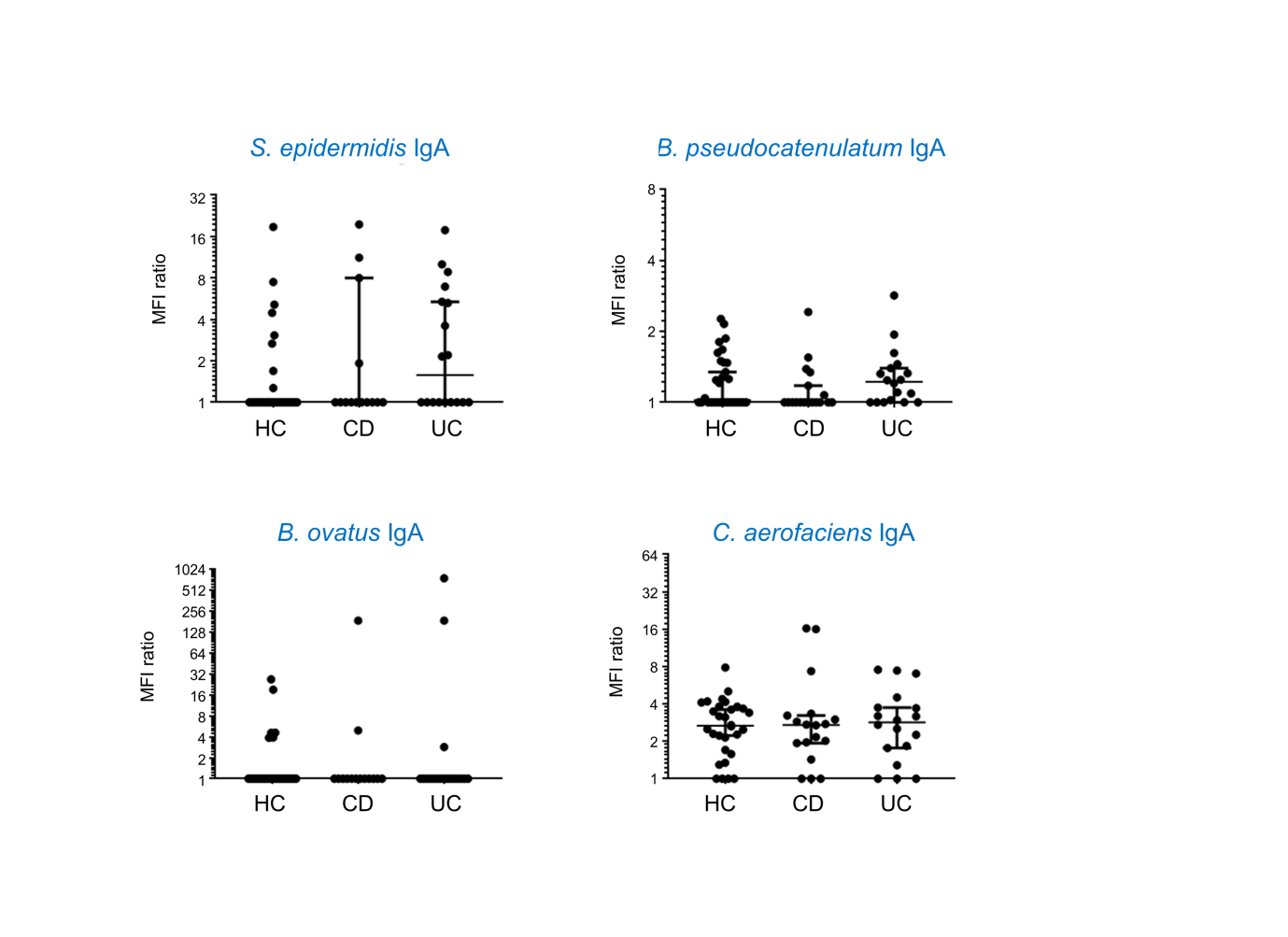


Figure S6. Circulating specific IgA antibodies to immunogenic commensal species in HC, CD and UC donors. Plasma was assayed for IgA antibodies using a coating assay. Ratios of median fluorescence intensity of anti-IgA-stained vs isotype control for each sample are shown (median and 95% CIs). Kruskal-Wallis tests were used to compare groups; n=30 HC, n=18 CD&UC. IgA responses to other species were undetectable.
